# Eravacycline susceptibility was impacted by genetic mutation of 30S ribosome subunits, and branched-chain amino acid transport system II carrier protein, Na/Pi cotransporter family protein in *Staphylococcus aureus*

**DOI:** 10.1186/s12866-020-01869-6

**Published:** 2020-07-01

**Authors:** Zhanwen Wang, Zhiwei Lin, Bing Bai, Guangjian Xu, Peiyu Li, Zhijian Yu, Qiwen Deng, Yongpeng Shang, Jinxin Zheng

**Affiliations:** 1 Department of Infectious Diseases and Shenzhen key laboratory for endogenous infections, Shenzhen Nanshan people’s Hospital and the 6th Affiliated Hospital of Shenzhen University Health Science Center, No 89, Taoyuan Road, Nanshan District, Shenzhen, 518052 China; 2 Quality control Center of Hospital Infection Management of Shenzhen, Shenzhen Nanshan People’s Hospital of Guangdong Medical University, No 89, Taoyuan Road, Nanshan district, Shenzhen, 518052 China

**Keywords:** Eravacycline, *Staphylococcus aureus*, Antimicrobial agent, Antimicrobial activity

## Abstract

**Background:**

Our previous research indicated the excellent in vitro antibacterial activity of Eravacycline (Erava) and its heteroresistance frequency against clinical *Staphylococcus aureus* isolates. In this study, we further aimed to investigate the mechanisms of Erava resistance and heteroresistance in *S. aureus*. Eight parental *S. aureus* isolates were induced under Erava pressure in vitro and the Erava-resistant isolates were selected and identified. Then, the genetic mutations of 30S ribosomal subunits were analyzed by PCR and sequence alignment. RT-qPCR analysis were performed to compare the relative expression of eight candidate genes impacting the susceptibility of tetracycline (Tet) between the resistant or heteroresistant and parental isolates. Furthermore, the in vitro overexpression vectors of three selected candidate genes were constructed to test their impact on the heteroresistance and resistance of Erava in *S. aureus*.

**Results:**

The MICs elevation in Erava-induced resistant *S. aureus* isolates were identified and the increasing MICs values of another two Tet class antibiotics, including both omadacycline (Omada) and tigecycline (Tige) were also tested. Genetic mutations in 30S ribosomal protein S10 were found frequently in Erava-derived resistant isolates. RT-qPCR analysis and the in vitro overexpression experiments indicated that *USA300HOU_RS00550* (an Na/Pi cotransporter family protein) and *USA300HOU_RS01625* (a branched-chain amino acid transport system II carrier protein) contributed to Erava heteroresistance in *S. aureus*.

**Conclusion:**

Genetic mutation of 30S ribosome subunits contributed to Erava resistance, and the transcriptional overexpression of *USA300HOU_RS01625* and *USA300HOU_RS00550* also participated in the occurrence of Erava heteroresistance in *S. aureus.*

## Background

*Staphylococcus aureus* is an important pathogen that leads to a wide spectrum of infectious diseases in humans, and the dissemination of methicillin-resistant *S. aureus* strains (MRSA) has posed a worldwide health threat in recent years [1, 2]. Recently, the emergence of multi-drug resistant *S. aureus*, including the vancomycin-intermediate or resistant, linezolid or daptomycin resistant isolates, has threatened the treatment options in clinics [3]. Thus, it’s urgent to develop new antimicrobials for the treatment of *S. aureus* infection.

Previous studies have indicated the high frequency of tetracycline resistance in *S. aureus* clinical isolates word wide [4, 5]. The dissemination of tetracycline resistance mechanism also limited the application of tetracycline derivatives, including minocycline and doxycycline, in the treatment choices of the *S. aureus* infection. Recently, the optimization of tetracycline class drugs promote the development of several new-generation tetracycline derivatives, such as tigecycline (Tige), omadacycline (Omada) and eravacycline (Erava), and multiple reports have also indicated the robust antimicrobial activity of these new-generation tetracycline derivatives against *S. aureus* and other gram positive bacteria [6–8]. Our previous research demonstrated the excellent in vitro antibacterial activity of eravacycline (Erava) and its heteroresistance frequency against *S. aureus* clinical isolates, however, the mechanism of Erava resistance and heteroresistance in *S. aureus* still need to be further studied [7].

High level of transcriptional expression of tetracycline specific resistance factors, such as *tet(M)* and *tet(K)*, have been found to mediate the Tige MIC creep in *E. faecium* isolates [8, 9]. Our previous data indicated that *tet(K)* was frequently detected in MSSA with Erava MIC ≥0.5mg/L and it’s still unknown whether *tet(K)* participated in the Erava heteroresistance or resistance [7]. The reduced susceptibility to Tige have been widely explained by the emergent mutations in genes encoding several 30S ribosome subunits, including 16S rRNA and 30S ribosome protein S10 [8–17]. The clinical significance of the genetic mutations of 30S ribosome subunits remains elusive should be further studied. Thus, the aim of this study was to examine the resistance mechanism in vitro by Erava-induced resistant *S. aureus*. The impact of the genetic mutations of 30S ribosome subunits on Erava susceptibility were also investigated. The transcriptional levels of eight candidated genes, which were previously reported to impact the Omada susceptibility, in the parental *S. aureus* isolates were compared with Erava heteroresistant and resistant derivative clones.

## Results

### Mechanism of Erava-induced resistance in *S. aureus* under Erava pressure

In order to investigate the relationship of Erava MICs creeps with the genetic mutations of 30S ribosome subunits, eight Erava susceptible *S. aureus* were selected in vitro under Erava pressure. The Erava resistant clones were identified and the MICs of Erava, Omada and Tige were measured as described in Table 1, indicating the cross resistance of Erava with Omada and Tige in these Erava-induced resistant *S. aureus* isolates. Genetic mutation of 30S ribosome subunits were determined in these Erava-induced resistant *S. aureus* isolates (Shown in Table 1), indicating the correlation of Erava MICs creep with the increasing copies numbers of five 16SrRNAs genes with genetic mutations. Whereas, the high frequency of T170G mutation in RR1, A1124G in RR2, C810T in RR3, G848C in RR4 and G1036A in RR5 was found in Erava-induced resistant *S. aureus.* The amino acid mutations Let47Let and Tyr87His in 30S ribosome protein S10 were found frequently in Erava-resistant *S. aureus* isolates. There were no mutations detected in the 30S ribosome protein S3 (data not shown). Importantly, increasing Erava MICs were accompanied by the increasing MICs values of both Omada and Tige in Erava-resistant *S. aureus* isolates, indicating that Erava cross-resistance with Omada and Tige can be induced under Erava pressure.

**Table 1:** The MICs determination and genetic mutations of tetracycline target sites in Erava-induced resistant *S. aureus* isolates

Strains	MICs values (mg/L)			Mutation of 30S ribosome subunits					
	Omada	Tige	Erava	RR1	RR2	RR3	RR4	RR5	S10
CHS221(parental strain)	0.5	0.5	0.25	W	W	W	W	W	W
CHS221-E1^a^	16	32	16	T170G	G77A; A1124G	C810T G848C	G185A; G1036A	W	W
CHS221-E2^a^	32	32	16	T170G	G77A; A1124G	G742A; C810T	G185A; G1036A	W	W
CHS165(parental strain)	0.5	0.5	0.25	W	W	W	W	W	W
CHS165-E1^a^	32	32	8	T170G; A972T	A1124G	C810T	G185A; G1036A	W	Tyr87His
CHS165-E2^a^	32	64	16	T170G G848C	A1124G	C810T	G185A; G1036A	G848A	Let47Let; Tyr87His
149(parental strain)	0.5	0.25	0.06	W	W	W	W	W	W
149-E1^a^	16	8	8	T170G;	G77A; A1124G	C810T	G185A; G1036A	W	Let47His;
149-E2^a^	32	32	32	T170G	G77A; AG847-848TA; A1124G	C810T	G185A; G1036A	W	Ile46Ile
CHS759(parental strain)	0.5	0.25	0.25	W	W	W	W	W	W
CHS759-E1^a^	16	8	8	T170G	A1124G	C810T	G1036A	W	W
CHS759-E2^a^	16	32	16	T170G	G77A; A1124G	C810T	G185A; G1036A	T1257C	W
CHS810(parental strain)	0.5	0.25	0.25	W	W	W	W	W	W
CHS810-E1^a^	16	32	8	T170G	A1124G	C810T	G1036A	W	Tyr87His
CHS810-E2^a^	16	32	8	T170G; G848C	G77A; A1124G	C810T	G1036A	W	Tyr87His
CHS820(parental strain)	0.5	1	0.125	W	W	W	W	W	W
CHS820-E1^a^	16	64	16	T170G	G77A; A1124G	C810T	G185A; G1036A	C1254T	Let47His
CHS820-E2^a^	16	64	16	T170G	G77A; A1124G	C810T	G185A; G1036A	C1254T; G1261C	Let47His
N315(parental strain)	0.5	0.5	0.25	W	W	W	W	W	W
N315-E1^a^	8	8	8	T170G; G1041A	A1124G	C810T; G848C	G185A	T846A	Tyr87His
N315-E2^a^	8	8	8	T170G; G1041A	A1124G	C810T	G185A	W	Tyr87His
MS4(parental strain)	0.5	0.25	0.25	W	W	W	W	W	W
MS4-E1^a^	16	32	16	T170G	A1124G	C810T	G1036A	W	Let47His

^a^: Erava-induced resistant isolates, respectively; RR1–5: five separate copies of the 16S rRNA gene; W, wild-type (no mutation)

### The transcriptional expression level of the candidate genes correlated with Erava susceptibility in *S. aureus*

The Erava MIC creep in heteroresistance clones could be previously reported to correlate with the Efflux pump, therefore we hypothesize that efflux pumps or membrane proteins might participate in the heteroresistance development. Our previous reported indicated the overexpression of eight candidate genes, including *USA300HOU_RS00705*, *USA300HOU_RS03535*, *USA300HOU_RS01625*, *USA300HOU_RS00550*, *USA300HOU_RS13205*, *USA300HOU_RS13945*, *USA300HOU_RS10505*, and *USA300HOU_RS00660*, might impact the Omada susceptibility in *S. aureus.* The profiles of the eight candidate genes were summarized in Table S1**.** Furthermore, high detection frequency of *tet(K)* and *tet(L)* in *S. aureus* with Erava MICs≥0.5mg/L, whereas whether the transcriptional level of *tet(K)* and *tet(L)* was correlated with Erava susceptibility need to be studied.

**Fig. 1:**
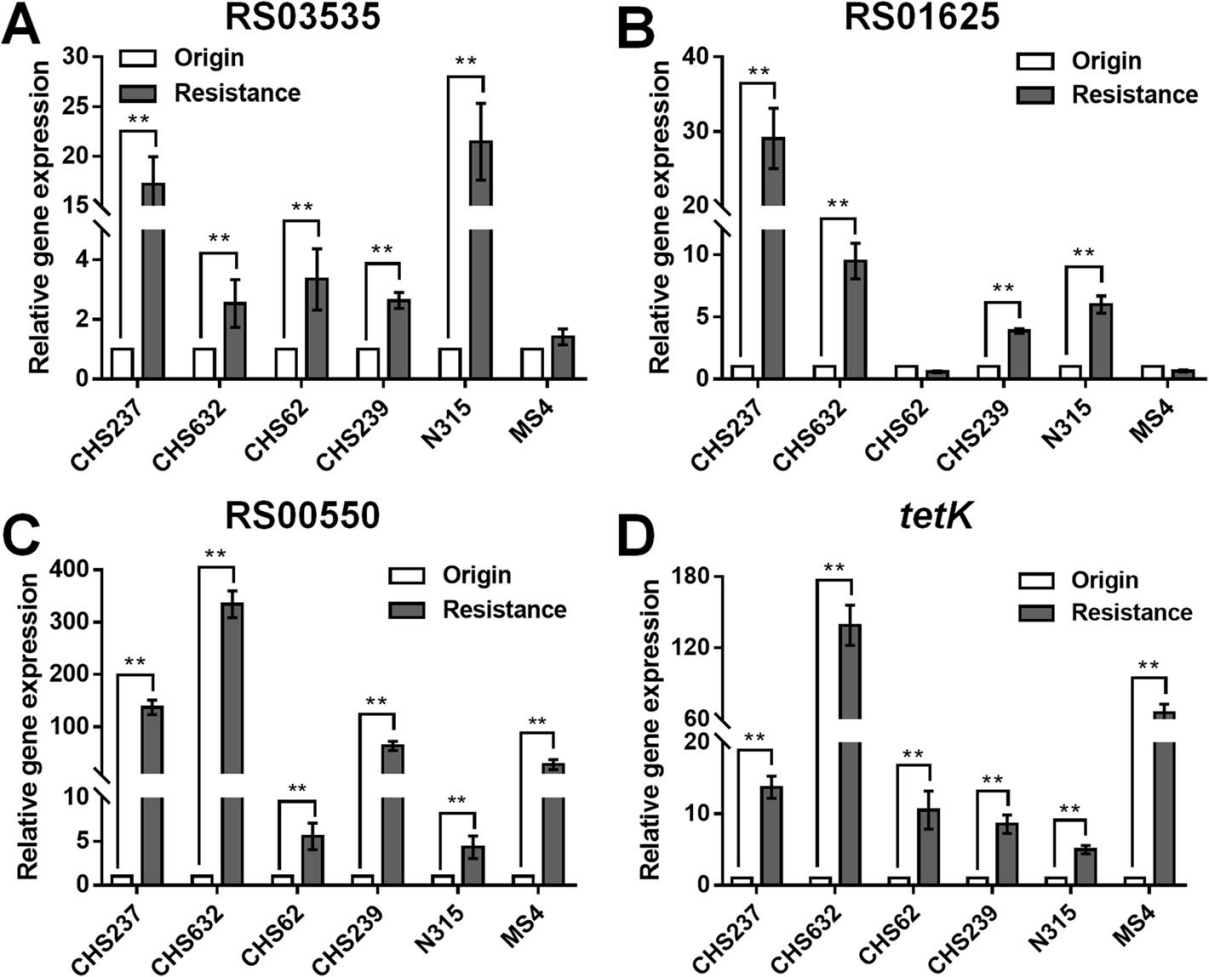
Significant elevation of the relative transcriptional expression level of the three candidate genes, including USA300HOU_RS03535, USA300HOU_ RS01625, USA300HOU_RS00550 and tetK in heteroresistance or Erava-resistant clones compared with that of the parental. The Origin represented the parental isolates including CHS237, CHS632, CHS62, CHS239, N315 and MS4; Resistance represented the corresponding heteroresistance isolates CHS237-E1, CHS632-E1, CHS62-E1, CHS239-E1, which was reported previously [6], and two Erava-resistant isolates shown in Table 1, including N315-E1 and MS4-E1. Relative expression of USA300HOU_RS03535 (**a**), USA300HOU_RS01625 (**b**), USA300HOU_RS00550 (**c**), tet(K) (**d**) were demonstrated by RT-qPCR analysis. The housekeeping gene *gyrB* was used as the endogenous reference gene. The original (parental) strain was used as the reference strain (expression = 1.0). All RT-qPCR were carried out in triplicate. **p < 0.01 (Student’s t-tests)

**Fig. 2:**
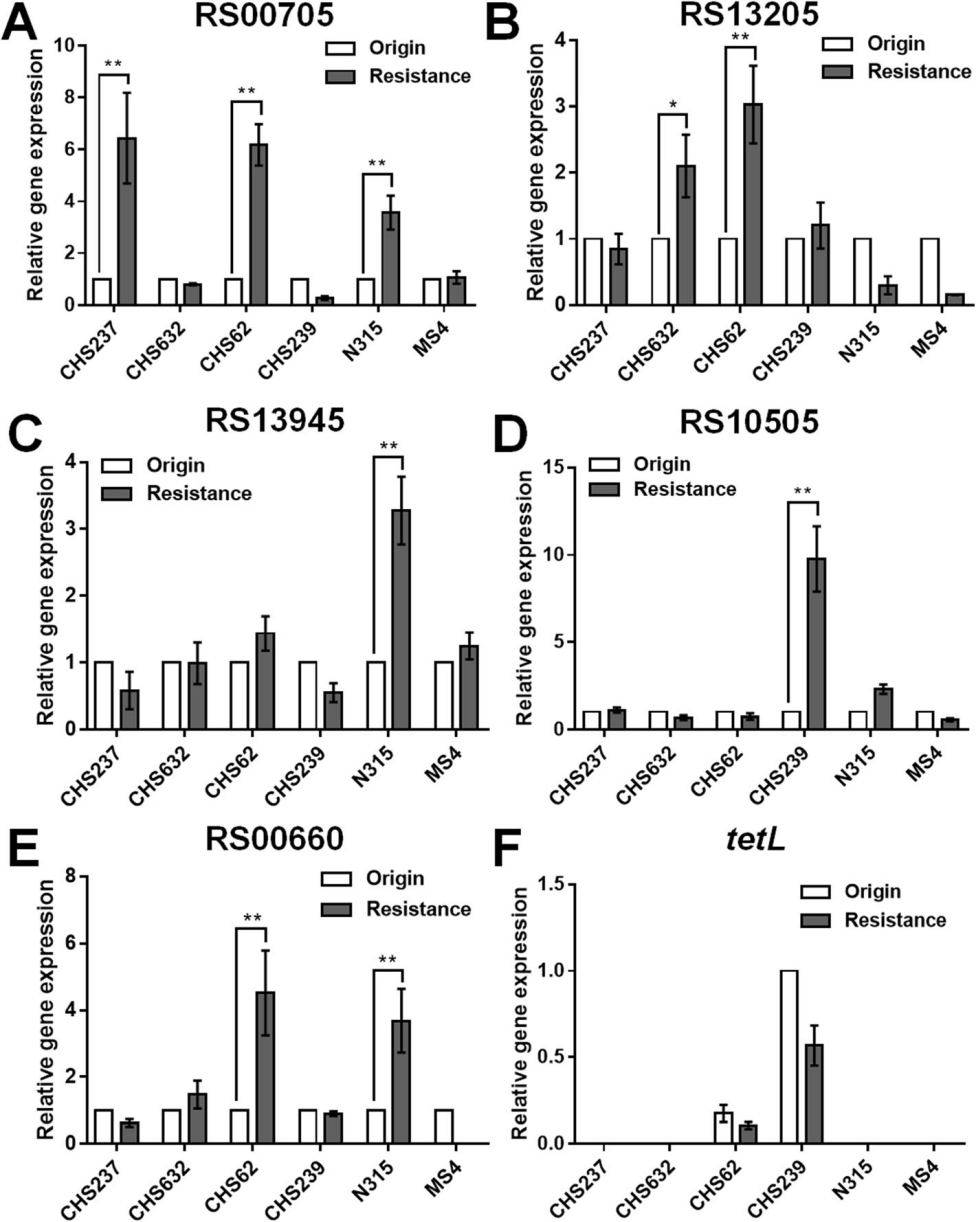
No significant elevation of the relative transcriptional expression level of the five candidate genes, including USA300HOU_RS00705, USA300HOU_RS13205, USA300HOU_RS13945, USA300HOU_RS10505, USA300HOU_RS00660 and tetL in heteroresistance or Erava-resistant clones compared with that of the parental. The origin represented the parental isolates including CHS237, CHS632, CHS62, CHS239, N315 and MS4; Resistance represented the corresponding heteroresistance isolates CHS237-E1, CHS632-E1, CHS62-E1, CHS239-E1, which was reported previously [6], and two Erava-resistant isolates shown in Table 1, including N315-E1 and MS4-E1. Relative expression of USA300HOU_RS00705 (**a**), USA300HOU_RS13205 (**b**), USA300HOU_RS13945 (**c**), USA300HOU_RS10505 (**d**), USA300HOU_RS00660(**e**), tet(L) (**f**), were demonstrated by RT-qPCR analysis. The housekeeping gene *gyrB* was used as the endogenous reference gene. The original (parental) strain was used as the reference strain (expression = 1.0). All RT-qPCR were carried out in triplicate. *p < 0.5; **p < 0.01 (Student’s t-tests)

We have previously reported the heteroresistance occurrence of the parental isolates, including CHS237, CHS632, CHS62, CHS239 and its corresponding heteroresistance isolates in which the genetic mutation of 30S ribosome subunits was not found. Furthermore, the relative transcriptional expression of these eight candidate genes, *tet(K)* and *tet(L)* were investigated by RT-qPCR analysis. The relative transcriptional levels of these genes were compared between these four parental isolates and their heteroresistance and between two parental isolates, N315 and MS4, and their Erava induced resistant isolates respectively. Interestingly, the transcriptional levels of *USA300HOU_RS03535*, *USA300HOU_ RS01625* (encodes a branched-chain amino acid transport system II carrier protein), *USA300HOU_RS00550* (encodes a Na/Pi cotransporter family protein) and *tet(K)* were shown with significant elevation in heteroresistance or Erava-resistant clones compared with that of the parental (Fig. 1). However, the other six candidate genes seemed no significant elevation (Fig. 2), suggesting these four candidate genes (*USA300HOU_RS03535*, *USA300HOU_RS01625*, *USA300HOU_RS00550* and *tet(K)*) might contribute to impact the Erava susceptibility and need to be further verified by the in vitro overexpression experiments.

### Relationship between three candidate genes overexpression and Erava susceptibility

The impacts of four candidate genes, including *USA300HOU_RS03535*, *USA300HOU_ RS01625*, *USA300HOU_RS00550* and *tet(K)*, were further tested by the in vitro overexpression experiments. The overexpression plasmids pRS00550, pRS01625, pRS03535, ptet(K) were successfully constructed and identified. Subsequently, the overexpression plasmids pRS00550, pRS01625, pRS03535, ptet(K) were transformed separately into clinical isolates SE4, SE7, SE13, CHS545 and CHS569, which have previously been reported with low expression level of three candidate genes [6]. The overexpression of the four genes in the five *S. aureus* isolates were confirmed by RT-qPCR and was shown in Fig. S1. The impact of the overexpression of these four candidate genes on Erava susceptibility was described in Table 2. Briefly to say, the protein encoded by these four candidate genes did not result in Erava MICs elevation, however, the transcriptional overexpression of *USA300HOU_RS00550* and *USA300HOU_RS01625* participate in the occurrence of Erava heteroresistance.

**Table 2:** Determination of Erava MIC value in the *S. aureus* derivatives with the overexpression of the three candidate genes and *tet(K)* and their impact on PAPs

Vectors	Strains	Erava MIC (mg/L)		PAP
		Parental isolates	Derivative isolates^**a**^	
**pRS00550**	SE7	0.25	0.125	+
	CHS545	0.125	0.125	+
	CHS569	0.125	0.125	+
**pRS01625**	SE4	0.125	0.125	+
	SE7	0.125	0.125	+
	SE13	0.125	0.125	+
	CHS545	0.125	0.125	+
	CHS569	0.0625	0.125	+
**pRS03535**	SE13	0.125	0.125	–
	CHS545	0.125	0.125	–
**ptetK**	SE7	0.125	0.125	–
	SE13	0.125	0.0625	–
	CHS545	0.25	0.125	–
	CHS569	0.125	0.125	–

*PAP* population analysis profiling

^a^: The overexpression of *USA300HOU_RS03535*, *USA300HOU_ RS01625*, *USA300HOU_RS00550* and *tet(K)* in *S. aureus* clinical isolates SE4, SE7, SE13, CHS545 and CHS569

## Discussion

Recent crystallographic analysis studies have revealed the same mechanism of the new- generation tetracycline class antibiotics, including Tige, Omada and Erava, to inhibit bacterial protein synthesis by binding to the 30S ribosomal subunits, including 16SrRNA and 30S ribosome protein S10 [8, 18]. Previous researches indicated that genetic mutations affecting the 30S ribosome subunits (i.e., 16SrRNA and ribosome proteins S10) have been shown to confer resistance to Tige and Omada in *S. aureus* [9–17, 18]. However, the characteristics of genetic mutation of the 30S ribosome subunits and cross resistance in Erava resistant *S. aureus* remains unknown. This study indicated that similar to other tetracycline class, Erava MIC elevation can be coupled with the increasing copies numbers of 16SrRNA genes with genetic mutations. Meanwhile, our study revealed high frequency of genetic mutations in 30S ribosome protein S10 in Erava-derived resistant isolates, indicating this protein might be an important factor in Erava resistance evolution. Previous multiple reports have shown that the mutation in 30S ribosome protein S10 of *S. aureus* can result in the MIC elevation of tetracycline, Omada and Tige, indicating its important role in the resistance evolution of tetracycline class drugs [6, 9, 12, 13, 15, 17]. Therefore, the cross resistance under Erava pressure with Omada and Tige might be mainly explained by the genetic mutation of 30S ribosome subunits

The frequent occurrence of antibiotics heteroresistance can result in the treatment failure. Our previous report indicated the occurrence of Erava heteroresistance in *S. aureus* and this phenomenon would enhance the difficulty for the treatment of *S. aureus* infection with Erava [7]. Our previous indicated the transcriptional expression level of the eight candidate genes, including *USA300HOU_RS00705*, *USA300HOU_ RS03535*, *USA300HOU_RS01625*, *USA300HOU_RS00550*, *USA300HOU_RS13205*, *USA300HOU_RS13945*, *USA300HOU_RS10505*, and *USA300HOU_RS00660*, which have been shown to be correlated with the Omada heteroresistance [6]. Our previous data indicated the cross resistance between Omada and Erava in *S. aureus* and therefore, whether these eight candidate genes might contribute to impact the Erava susceptibility should be further evaluated. Although the high detection frequency of *tet(K)* and *tet(L)* in *S. aureus* was reported in our previous study [7], it remained unknown that whether the overexpression of *tet(K)* and *tet(L)* would impact the Erava susceptibility. Our data indicated that the overexpression of *USA300HOU_RS00550* and *USA300HOU_RS01625* would enhance the occurrence of Erava heteroresistance but have no direct impact on Erava MIC creep, demonstrating their overexpression can result in Erava MIC elevation under Erava pressure in vitro. Our previous phylogenic analysis have showed that both *USA300HOU_RS00550* and *USA300HOU_ RS01625* encode efflux pump family proteins [6, 7], supporting our hypothesis that efflux pump or membrane proteins contribute to Erava or Omada heteroresistance and the cross- resistance between Erava and Omada in *S. aureus* should arouse our attention.

## Conclusions

In conclusion, Erava susceptibility in *S. aureus* was impacted by genetic mutation of 30S ribosome subunits, including 16SrRNA and 30S ribosome protein S10. Furthermore, two efflux pump family proteins encoded by *USA300HOU_RS01625* (a branched-chain amino acid transport system II carrier protein) and *USA300HOU_RS00550* (an Na/Pi cotransporter family protein) contribute to Erava heteroresistance in *S. aureus*.

## Methods

### Bacterial isolates

The *S. aureus* clinical isolates used in this study were collected from the different inpatients of Shenzhen Nanshan People’s Hospital (a tertiary hospital with 1200 beds) between 2010 and 2016. The in vitro induction *S. aureus* clinical isolates, including CHS221, CHS165, 149, CHS759, CHS810, CHS820, have been used in our previous study [6]. N315 (GenBank accession number: BA000018) and MS4 (GenBank accession number: CP009828) are two reference *S. aureus* isolates reserved in our lab, which are MRSA and sensitive to Tige, Omada. The parental MSSA isolates CHS237, CHS632, CHS62, CHS239 and their heteroresistant derived clones CHS237-E1, CHS6 32-E1, CHS62-E1, CHS239-E1, included in this study, have been described in our previous research for the investigation of Erava heteroresistance [7]. The *S. aureus* isolates, including SE4, SE7, SE13, CHS545 and CHS569, were used as reference isolates for the overexpression experiments as described previously [6]. The minimum inhibitory concentration (MIC) of antimicrobials and multilocus sequence typing of the isolates used in this study were determined in our previous research [7], and were summarized in Table S2.

All procedures involving human participants were performed in accordance with the ethical standards of Shenzhen University and the 1964 Helsinki declaration and its later amendments, or comparable ethical standards. For this type of study, formal consent is not required.

### Antimicrobial susceptibility and population analysis profiling (PAP) development

Omada, Tige and Erava were purchased from the Medicines Company (Med Chem Express, Monmouth Junction, NJ) and their MICs were determined by the agar dilution method according to CLSI guidelines [19]. Because the CLSI guidelines provide no recommendation for the Erava MIC susceptibility breakpoints against *S. aureus*, we adopted an MIC susceptibility breakpoint of 0.5 mg/L, the value recommended for tigecycline nonsusceptibility in Gram-positive bacteria and defined heteroresistance as growth in 0.5mg/L Erava [7, 20]. PAP experiments were conducted as previously [21, 22]. Briefly, 50μL aliquots (~ 10^8^ colony forming units/ml) were spread onto Müller-Hinton agar plates containing serially diluted Erava (0.5, 1.0, 2.0, 3.0 mg/L). Colonies were counted after 24h of incubation at 37°C. Erava heteroresistance was defined as the observation of subpopulations isolated from the Erava-containing plates able to grow in the presence of 0.5mg/L Erava (detection limit, ≥ 5 colony forming units/plate).

### Polymerase chain reaction (PCR) and sequence alignment

Genomic DNA of all clinical isolates was extracted and used as templates for PCR amplification in lysis buffer for microorganisms to direct PCR (Takara Bio Inc., Japan). The presence of 30S ribosomal subunit mutations, including five separate copies (RR1, RR2, RR3, RR4, RR5) of the 16S rRNA gene, the genes encoding the 30S ribosomal proteins S3 and S10, were analyzed by PCR and sequence alignment as described previously [6], and the PCR primers were listed in Table S3.

### In vitro induction of Erava resistance

Eight parental *S. aureus* isolates, including six clinical isolates (MSSA: CHS221, CHS165, 149; MRSA: CHS759, CHS810, CHS820) and two reference strains (MRSA: N315 and MS4), were used to select Erava-resistant isolates in vitro as described previously [6]. Briefly, these isolates were subcultured serially in Mueller-Hinton broth containing gradual increasing Erava concentrations with the initial concentration being MIC followed by successive increases to 2×, 4×, 8×, 16×, 32×MICs [23], with four passages at each concentration. Isolates from the passages of each concentration were stored at -80°C in Mueller-Hinton broth containing 40% glycerol for further analysis, including Tet-target site genetic mutation detection and MICs determination.

### Quantitative real time PCR (RT-qPCR) analysis

Total RNA of *S. aureus* isolates was extracted with a RNeasy mini kit (QIAGEN GmbH, Hilden, Germany) and RT-qPCR was performed with an SYBR Premix Ex Taq II kit in a Mastercycler EP Realplex system (Eppendorf, Hamburg, Germany) according to the manufacturer’s instructions (Takara Bio Inc., Shiga, Japan). The internal control gene *gyrB* was used to normalize gene expression. Threshold cycle (Ct) numbers were determined by detection system software and analyzed with the 2^−△△Ct^ method. The RT-qPCR primers used were described previously and as shown in Table S4 [6]. RT-qPCR was performed in triplicate at least three times.

### Gene overexpression

The overexpression vectors pRS00550, pRS01625, pRS03535, ptetK were constructed by integrating the candidate gene fragments, including *USA300HOU_RS00550* (encodes a Na/Pi cotransporter family protein), *USA300HOU_RS01625* (encodes a branched-chain amino acid transport system II carrier protein), *USA300HOU_RS03535* and *USA300HOU_Tet(K)*, each into separate pIB166 vectors as described previously [6]. Then, the vectors were transformed separately each into three to five Erava-sensitive isolates and confirmed by PCR and Sanger sequencing. The overexpression was induced by 2 μM CdCl_2_ and verified by RT-qPCR. All strains, plasmids, and primers used for overexpression analysis are listed in Tables S5 and S6.

### Statistical analysis

Continuous data were analyzed by Student’s t-tests with SPSS software package (version 17.0, Chicago, IL). P values < 0.05 were regarded as statistically significant.

## Supplementary information

**Additional file 1 Table S1** The profiles of the eight candidate genes correlated with eravacycline susceptibility in *S. aureus*.

**Additional file 2 Table S2** MICs values and MLST of the *S. aureus* isolates used in this study.

**Additional file 3 Table S3** Primers used for the detection of Tet-resistance genes and Tet target sites in *S. aureus* by PCR.

**Additional file 4 Table S4** Primers used for RT-qPCR in this study.

**Additional file 5 Table S5** Strains and plasmids used for the overexpression test in this study.

**Additional file 6 Table S6** PCR primers used for the overexpression vector constructions in this study.

**Additional file 7 Figure S1** Overexpression of USA300HOU_RS00550, USA300HOU_RS01625, USA300HOU_RS03535 and tetK in different Eravacycline-sensitive clinical *S. aureus* isolates. The RNA levels of *USA300HOU_RS00550* (A), *USA300HOU_RS01625* (B), *USA300HOU_RS03535* (C) and *tetK* (D) were determined by qRT-PCR. The wild-type isolates were used as the reference strain (mRNA level = 1.0).

## Data Availability

All data generated or analyzed during this study are included in this published article [and its supplementary information files].

## Abbreviations

MRSA: Methicillin-resistant *S. aureus*;
MSSA: Methicillin-sensitive *S. aureus*
PAP: Population analysis profiling
MIC: Minimal inhibitory concentration
RT-qPCR: Quantitative real time PCR
