# Supplementary material for: Eravacycline susceptibility was impacted by genetic mutation of 30S ribosome subunits, and branched-chain amino acid transport system II carrier protein, Na/Pi cotransporter family protein in Staphylococcus aureus
Source: BMC Microbiol. 2020 Jul 1;20:189. doi: 10.1186/s12866-020-01869-6 (PMC7329441; doi:10.1186/s12866-020-01869-6)
Supplement: Supplementary file 1 — Additional file 1 Table S1 The profiles of the eight candidate genes correlated with eravacycline susceptibility in S. aureus. [file 12866_2020_1869_MOESM1_ESM.docx]

**Table S1** The profiles of the eight candidate genes correlated with eravacycline susceptibility in *S. aureus*.

| **Gene_ID** | **Description/Function** |
| --- | --- |
| ***USA300HOU_RS00705*** | Cell wall-anchored protein SasD |
| ***USA300HOU_RS03535*** | Membrane protein |
| ***USA300HOU_RS01625*** | Branched-chain amino acid transport system II carrier protein |
| ***USA300HOU_RS00550*** | Na/Pi cotransporter family protein |
| ***USA300HOU_RS13205*** | Amino acid permease |
| ***USA300HOU_RS13945*** | PTS transporter subunit IIC |
| ***USA300HOU_RS10505*** | hypothetical protein |
| ***USA300HOU_RS00660*** | MFS transporter |
