# Supplementary material for: Eravacycline susceptibility was impacted by genetic mutation of 30S ribosome subunits, and branched-chain amino acid transport system II carrier protein, Na/Pi cotransporter family protein in Staphylococcus aureus
Source: BMC Microbiol. 2020 Jul 1;20:189. doi: 10.1186/s12866-020-01869-6 (PMC7329441; doi:10.1186/s12866-020-01869-6)
Supplement: Supplementary file 2 — Additional file 2 Table S2 MICs values and MLST of the S. aureus isolates used in this study. [file 12866_2020_1869_MOESM2_ESM.docx]

**Table S2** MICs values and MLST of the *S. aureus* isolates used in this study.

| **Isolates** | **MIC (mg/L)** | | | | | | | | **MLST** |
| --- | --- | --- | --- | --- | --- | --- | --- | --- | --- |
|  | **Lin** | **Van** | **Amp** | **Oxa** | **Ery** | **Omada** | **Erava** | **Tig** |  |
| **CHS221** | 2 | ≤1 | ≤2 | 0.5 | >8 | 0.5 | 0.25 | 0.5 | ST7 |
| **CHS165** | 1 | ≤1 | >8 | 0.5 | >8 | 0.5 | 0.25 | 0.5 | ST398 |
| **149** | 2 | ≤1 | ≤2 | 0.5 | >8 | 0.5 | 0.06 | 0.25 | ST7 |
| **CHS759** | 2 | ≤1 | >8 | >8 | >8 | 0.5 | 0.25 | 0.25 | ST239 |
| **CHS810** | 2 | ≤1 | >8 | >8 | >8 | 0.5 | 0.25 | 0.25 | ST59 |
| **CHS820** | 2 | ≤1 | >8 | >8 | >8 | 0.5 | ≤0.125 | 1 | ST239 |
| **CHS237** | 2 | ≤1 | ≤2 | 0.5 | >8 | 0.5 | 0.5 | 0.5 | ST398 |
| **CHS632** | 2 | ≤1 | ≤2 | ≤0.25 | >8 | 0.5 | 0.5 | 0.25 | NT |
| **CHS62** | 2 | ≤1 | ≤2 | 0.5 | >8 | 0.5 | 0.5 | 0.25 | ST398 |
| **CHS239** | 2 | ≤1 | ≤2 | 0.5 | >8 | 0.5 | 0.5 | 0.5 | ST398 |
| **SE4** | 2 | ≤1 | ≤2 | 0.5 | ≤0.25 | 0.5 | 0.125 | 0.25 | ST7 |
| **SE7** | 2 | ≤1 | ≤2 | 0.5 | ≤0.25 | 0.5 | 0.125 | 0.25 | ST6 |
| **SE13** | 2 | ≤1 | ≤2 | 0.5 | ≤0.25 | 0.5 | 0.125 | 0.25 | NT |
| **CHS545** | 2 | ≤1 | 8 | >8 | ≤0.25 | 0.25 | 0.125 | 0.5 | NT |
| **CHS569** | 2 | ≤1 | ≤2 | >8 | ≤0.25 | 0.25 | 0.125 | 0.5 | ST3191 |
| **MS4** | 1 | ≤1 | >8 | >8 | ≤0.25 | 0.5 | 0.25 | 0.25 | ST59 |
| **N315** | 2 | ≤1 | >8 | >8 | >4 | 0.5 | 0.25 | 0.5 | ST5 |

Note: MIC, minimum inhibitory concentration; Lin, linezolid; Van, vancomycin; Amp, ampicillin; Oxa, oxacillin; Ery, erythromycin; Omada, omadacycline; Erava, eravacycline; Tig, tigecycline; MLST, multilocus sequence typing; NT, not typed;
