# Supplementary material for: Eravacycline susceptibility was impacted by genetic mutation of 30S ribosome subunits, and branched-chain amino acid transport system II carrier protein, Na/Pi cotransporter family protein in Staphylococcus aureus
Source: BMC Microbiol. 2020 Jul 1;20:189. doi: 10.1186/s12866-020-01869-6 (PMC7329441; doi:10.1186/s12866-020-01869-6)
Supplement: Supplementary file 3 — Additional file 3 Table S3 Primers used for the detection of Tet-resistance genes and Tet target sites in S. aureus by PCR. [file 12866_2020_1869_MOESM3_ESM.docx]

**Table S3** Primers used for the detection of Tet-resistance genes and Tet target sites in *S. aureus* by PCR.

| **Target gene** | **Primers** | **Sequence (5'-3')** | **Amplicon size (bp)** | **Reference** |
| --- | --- | --- | --- | --- |
| 16SrRNA-RR1 | RR1-F | ATATGTCACGTTATTCCGCATCTTC | 2086 | Bai B *et al*. 2019 |
|  | RR1-R | GCGGTGTTTTGAGAGATTATTTA |  |  |
| 16SrRNA-RR2 | RR2-F | ATATGTCACGTTATTCCGCATCTTC | 2075 | Bai B *et al*. 2019 |
|  | RR2-R | GCAGACGCACAGGACTTC |  |  |
| 16SrRNA-RR3 | RR3-F | ATATGTCACGTTATTCCGCATCTTC | 1936 | Bai B *et al*. 2019 |
|  | RR3-R | GTCGTCAAACGGCACTAATA |  |  |
| 16SrRNA-RR4 | RR4-F | ATATGTCACGTTATTCCGCATCTTC | 1756 | Bai B *et al*. 2019 |
|  | RR4-R | ATCACCCGCTCCATAGATAAT |  |  |
| 16SrRNA-RR5 | RR5-F | ATATGTCACGTTATTCCGCATCTTC | 2345 | Bai B *et al*. 2019 |
|  | RR5-R | AGGTGCGATGGCAAAACA |  |  |
| SA30S-S3 | S3-R | GCAGATTCGATTTGACGAGAT | 810 | Bai B *et al*. 2019 |
|  | S3-F | ACGGTAAAGAAGAAGCTAAAG |  |  |
| SA30S-S10 | S10-R | CTCGAAAATAGTTGAACTGACTAAG | 1920 | Bai B *et al*. 2019 |
|  | S10-F | TTCAGAAGATTTCTCAGTGATTACG |  |  |

Reference: Bai B, Lin Z, Pu Z *et al*. In vitro Activity and Heteroresistance of Omadacycline Against Clinical *Staphylococcus aureus* Isolates From China Reveal the Impact of Omadacycline Susceptibility by Branched-Chain Amino Acid Transport System II Carrier Protein, Na/Pi Cotransporter Family Protein, and Fibronectin-Binding Protein. *Front Microbiol*. 2019;10: 2546.
