# Supplementary material for: Eravacycline susceptibility was impacted by genetic mutation of 30S ribosome subunits, and branched-chain amino acid transport system II carrier protein, Na/Pi cotransporter family protein in Staphylococcus aureus
Source: BMC Microbiol. 2020 Jul 1;20:189. doi: 10.1186/s12866-020-01869-6 (PMC7329441; doi:10.1186/s12866-020-01869-6)
Supplement: Supplementary file 4 — Additional file 4 Table S4 Primers used for RT-qPCR in this study. [file 12866_2020_1869_MOESM4_ESM.docx]

**Table S4** Primers used for RT-qPCR in this study.

| **Primer** | **Primer sequence (5'-3')** | **Amplicon size (bp)** |
| --- | --- | --- |
| ***gyrB-F*** | ACATTACAGCAGCGTATTAG | 111 |
| ***gyrB-R*** | CTCATAGTGATAGGAGTCTTCT |  |
| ***RS00550-F*** | TCCAGTATTAGGTGTTATTG | 153 |
| ***RS00550-R*** | GTTGTTCCGATATTAGCA |  |
| ***RS01625-F*** | AATATGTGGATTGGTATGC | 113 |
| ***RS01625-R*** | ATACGATTGCCTACACTT |  |
| ***RS03535-F*** | TGCCTCAATTCCTACATT | 190 |
| ***RS03535-R*** | ATAACCTGCTACCATCATT |  |
| ***tetK-F*** | ACTGATTATGGTGGTTGTAG | 162 |
| ***tetK-R*** | ATAGGAAGTATAAGTAGGTAAGAC |  |
