# Supplementary material for: Eravacycline susceptibility was impacted by genetic mutation of 30S ribosome subunits, and branched-chain amino acid transport system II carrier protein, Na/Pi cotransporter family protein in Staphylococcus aureus
Source: BMC Microbiol. 2020 Jul 1;20:189. doi: 10.1186/s12866-020-01869-6 (PMC7329441; doi:10.1186/s12866-020-01869-6)
Supplement: Supplementary file 5 — Additional file 5 Table S5 Strains and plasmids used for the overexpression test in this study. [file 12866_2020_1869_MOESM5_ESM.docx]

**Table S5** Strains and plasmids used for the overexpression test in this study.

| **Strains or plasmids** | **Description** | **Source** |
| --- | --- | --- |
| ***S. aureus* strains** |  |  |
| SE4 | Erava-sensitive clinical *S. aureus* isolate | This study |
| SE7 | Erava-sensitive clinical *S. aureus* isolate | This study |
| SE13 | Erava-sensitive clinical *S. aureus* isolate | This study |
| CHS545 | Erava-sensitive clinical *S. aureus* isolate | This study |
| CHS569 | Erava-sensitive clinical *S. aureus* isolate | This study |
| **Plasmids** |  |  |
| pIB166 | *E. coli* (Cm^20^)-*Streptococcus* (Cm^20^) shuttle vector | Jingren Zhang, Tsinghua University |
| pRS00550 | pIB166 with insertion of the gene *USA300HOU_RS00550* | This study |
| pRS01625 | pIB166 with insertion of the gene *USA300HOU_RS01625* | This study |
| pRS03535 | pIB166 with insertion of the gene *USA300HOU_RS03535* | This study |
| ptet(K) | pIB166 with insertion of the gene *tet(K)* | This study |
