# Supplementary material for: Eravacycline susceptibility was impacted by genetic mutation of 30S ribosome subunits, and branched-chain amino acid transport system II carrier protein, Na/Pi cotransporter family protein in Staphylococcus aureus
Source: BMC Microbiol. 2020 Jul 1;20:189. doi: 10.1186/s12866-020-01869-6 (PMC7329441; doi:10.1186/s12866-020-01869-6)
Supplement: Supplementary file 6 — Additional file 6 Table S6 PCR primers used for the overexpression vector constructions in this study. [file 12866_2020_1869_MOESM6_ESM.docx]

**Table S6** PCR primers used for the overexpression vector constructions in this study.

| **Primers** | **Sequences（5’-3’）** | **Product**  **Length (bp)** | **Underline^a^** |
| --- | --- | --- | --- |
| **Construction of the overexpression plasmids** | | | |
| ORS00550-F | CGCGGATCCGATTTCAAATCATGAGACTGG | 2123 | BamHI |
| ORS00550-R | CCGGAATTCCTTACCGCATACTAATGAAGC |  | EcoRI |
| ORS00705-F | CGCGGATCCTGTGTAATGGGATAGCACGTA | 1108 | BamHI |
| ORS00705-R | CCGGAATTCTGCCAATATAACATTAGCGAG |  | EcoRI |
| ORS01625-F | CGCGGATCCTAGCAGTCCTCACTCATACAA | 1653 | BamHI |
| ORS01625-R | CCGGAATTCTAGACAGACGGTATCACTGAA |  | EcoRI |
| ORS03535-F | CGCGGATCCGTCATTGAACGTGATAATGTG | 1281 | BamHI |
| ORS03535-R | CCGGAATTCCCCGATAAGAGTAGAGACGAA |  | EcoRI |
| OtetK-F | CGCGGATCCTTTAGTGTATTAAATGAAATGGT | 1326 | BamHI |
| OtetK-R | CCGGAATTCCTATTCAAACTGCTTTTCAGAACG |  | EcoRI |
| **Verification of the overexpression plasmids** | | | |
| IDRS00550-F | TTCGCATTACAGAAGATGCAC | 828 |  |
| IDRS00550-R | CGCCTGTCACTTTGCTTGATA |  |  |
| IDRS00705-F | CGAATGTAGATGCAAATGGTG | 749 |  |
| IDRS00705-R | GCGCCTGTCACTTTGCTTGAT |  |  |
| IDRS01625-F | CCGTGTCGATTGCACTTGTAC | 803 |  |
| IDRS01625-R | CTTGTTGCTGTTCCTGTTCTG |  |  |
| IDRS03535-F | TGCCTCAATTCCTACATTGAC | 730 |  |
| IDRS03535-R | CGCCTGTCACTTTGCTTGATA |  |  |
| IDtetK-F | TGCTTCTGGAATGAGTTTGCT | 619 |  |
| IDtetK-R | GTTTCCTGCTAAACCATTTAG |  |  |

^a^Underlined sequences represent the restriction enzyme sites of primers;
