# Supplementary figures and images for: Eravacycline susceptibility was impacted by genetic mutation of 30S ribosome subunits, and branched-chain amino acid transport system II carrier protein, Na/Pi cotransporter family protein in Staphylococcus aureus
Source: BMC Microbiol. 2020 Jul 1;20:189. doi: 10.1186/s12866-020-01869-6 (PMC7329441; doi:10.1186/s12866-020-01869-6)

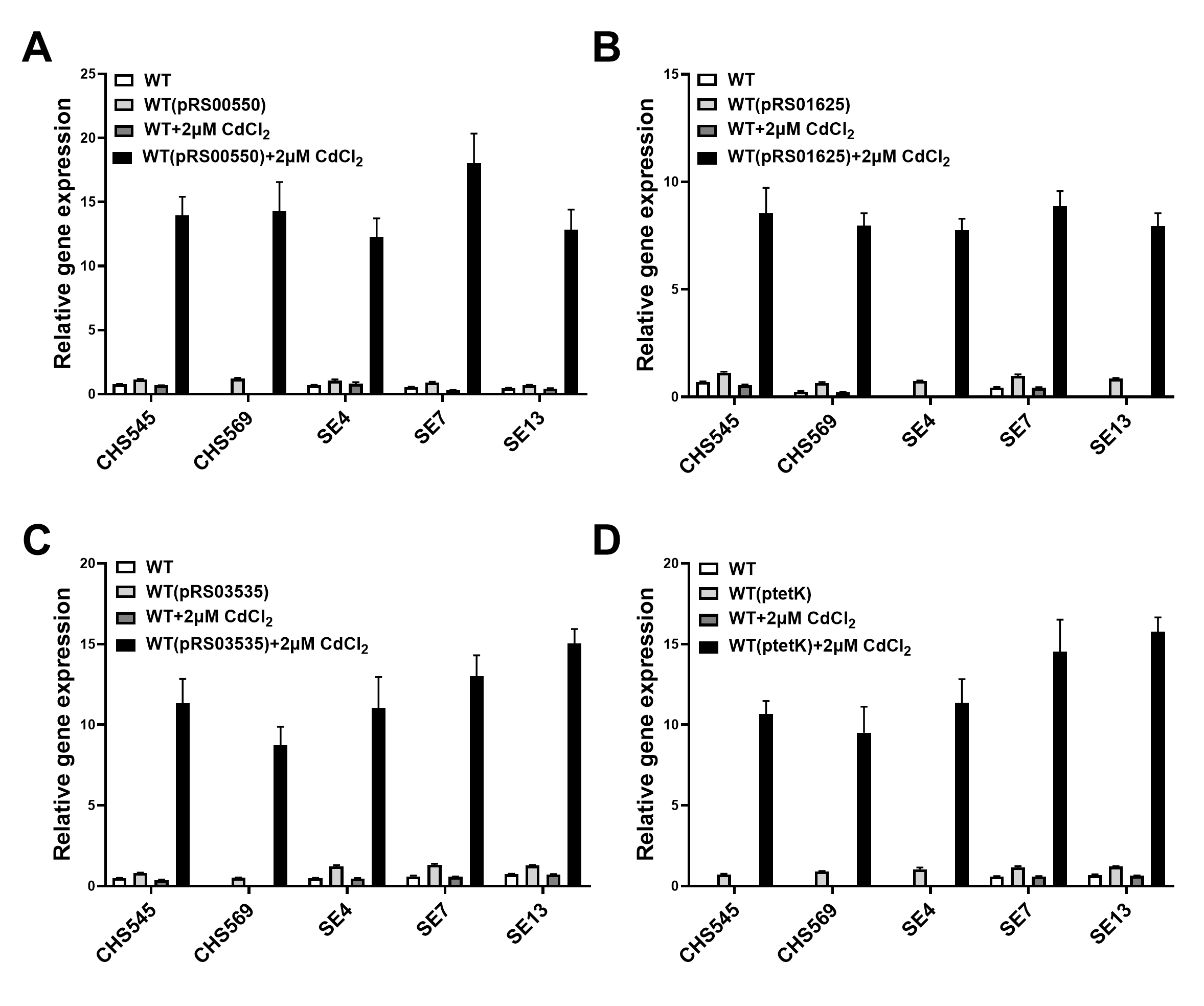

Supplement: Supplementary file 7 — Additional file 7 Figure S1 Overexpression of USA300HOU_RS00550, USA300HOU_RS01625, USA300HOU_RS03535 and tetK in different Eravacycline-sensitive clinical S. aureus isolates. The RNA levels of USA300HOU_RS00550 (A), USA300HOU_RS01625 (B), USA300HOU_RS03535 (C) and tetK (D) were determined by qRT-PCR. The wild-type isolates were used as the reference strain (mRNA level = 1.0). [file 12866_2020_1869_MOESM7_ESM.tif]
